# *Editorial Commentary:* Understanding BCG Is the Key to Improving It

**DOI:** 10.1093/cid/cit793

**Published:** 2013-12-13

**Authors:** Helen McShane

**Affiliations:** The Jenner Institute, University of Oxford, United Kingdom

**Keywords:** BCG, vaccine, efficacy

**(See the Major Article by Mangtani et al on pages 470–80.)**

The need for improved tuberculosis control remains a global health priority. The most cost-effective long-term solution for any infectious disease epidemic is effective vaccination. The only licensed vaccine against tuberculosis, BCG, when administered at birth, is highly effective at preventing disseminated disease in childhood. However, the protection conferred against pulmonary disease is highly variable, and a more effective and consistent vaccination regimen is urgently needed [1]. Leading approaches to developing a better tuberculosis vaccine include boosting BCG with a subunit vaccine incorporating one or several antigens from *Mycobacterium tuberculosis* in a potent antigen delivery system, and developing a recombinant strain of BCG (or another whole mycobacterial vaccine) to replace BCG with a safer and more effective vaccine [2]. The recent failure of the MVA85A trial to enhance efficacy in BCG-vaccinated South African infants suggests that improving on BCG-induced protection, at least in infants, may not be easy [3]. One of the challenges is the underlying variability in BCG efficacy against pulmonary disease, across different geographical areas and different age groups. Understanding the underlying mechanisms for this variability is important, both to optimize the delivery of BCG (or newer BCG replacement vaccines) and to facilitate the development of booster vaccines that overcome this variability.

Over the last decade or so, many different hypotheses have been proposed to explain the variability in efficacy observed in different clinical trials. These include differences in BCG and *M. tuberculosis* strains, host genetics, nutrition, coinfection with helminths, and exposure to nontuberculous mycobacteria (NTM). Whereas the relative importance of these different mechanisms may differ by geographical area, and more than one explanation may be involved, there is increasing evidence for a role of exposure to nontuberculous mycobacteria in explaining at least some of the variability. Two potentially complementary mechanisms have been proposed to explain how exposure to NTM might interfere with BCG efficacy: masking and blocking. The masking hypothesis is best illustrated by elegant work by Black and colleagues, where anti-mycobacterial immunity in BCG-naive adolescents in the United Kingdom and Malawi was evaluated, prior to and after BCG vaccination [4]. In the United Kingdom, baseline immunity was very low and there was a significant rise in antimycobacterial immunity after BCG vaccination. In contrast, in Malawi, baseline, prevaccination immunity was high. This was thought to be induced by NTM exposure as subjects with *M. tuberculosis* exposure had been excluded. Incremental rise in antimycobacterial immunity after BCG vaccination was much lower in these African adolescents, suggesting that the NTM induced immunity “masks” the effect of BCG vaccination, and that this preexisting immunity cannot be boosted with BCG. The “blocking” hypothesis suggests a more active immunological mechanism whereby the preexisting antimycobacterial immunity induced by NTM “blocks” the replication of BCG and therefore inhibits any protective effect. BCG is a live attenuated vaccine and efficacy is dependent on replication. In mice, preexposure to NTM can inhibit the protective effect of BCG, but interestingly, preexposure to NTM did not affect the efficacy of a (nonreplicating) subunit vaccine, a finding that is encouraging for the development of subunit booster vaccines [5].

In this issue of *Clinical Infectious Diseases*, Mangtani and colleagues provide further corroborating evidence for a role for NTM exposure in explaining the variability in BCG efficacy. They conducted a systematic review of all reported BCG efficacy trials, and examined associations with efficacy including immunological evidence of mycobacterial exposure prior to vaccination and BCG strain. This analysis was restricted to the most methodologically rigorous trials, with the caveat that many of these trials were conducted many decades ago (and most papers were published before 1973), in an era with different standards for statistical and methodological rigor. The authors find that the greatest average efficacy of BCG was when BCG was administered to neonates (rate ratio [RR], 0.41; 95% confidence interval [CI], .29–.58]) or when BCG was administered to school-age children with stringent tuberculin testing (as in the British Medical Research Council study in the 1950s [6]) (random-effects RR, 0.26; 95% CI, 0.18–.37). They confirm previous findings that efficacy correlates with latitude and increases with increasing trial site distance from the equator. In a univariate analysis, these 2 factors (distance from the equator and age at vaccination/tuberculin testing stringency) explained most of the between-trial variability in efficacy; using a 2-variable meta-regression model, these 2 factors explained all of the between-trial variability. Importantly, the authors also find that BCG strain does not explain the variability in BCG efficacy. This analysis also confirmed previous findings of a strong protective effect of BCG against meningeal or miliary disease. Although the authors are rightly cautious with their interpretation of these findings, given the large number of variables used in the multivariable analyses (7) compared with the number of studies (18), these findings add further support to existing evidence from human and animal studies that prior NTM exposure interferes with the efficacy of BCG, and that BCG is most effective in mycobacterially naive hosts.

If this theory is correct, there are some important practical implications. We should optimize deployment of BCG to administration as close to birth as possible. We know from studies conducted in The Gambia that exposure to NTM can happen very early in life and that withholding BCG until 4 months of age results in measurable antimycobacterial immunity, presumably induced by NTM [7]. Although at 9 months, antimycobacterial immunity was comparable between infants vaccinated either at birth or at 4 months in this Gambian study, we do not know which aspects of immunity correlate with protection, and it would be prudent to recommend BCG vaccination as soon as possible after birth. However, this recommendation cannot be applied in areas with high human immunodeficiency virus (HIV) prevalence, as excluding HIV infection prior to BCG is recommended in those regions. Just as important, this theory suggests that the effects of prior NTM exposure on candidate tuberculosis vaccines in development should also be evaluated. The efficacy of the live BCG replacement vaccines may also be affected by prior exposure to NTM, in the same way as BCG. The efficacy of subunit, nonreplicating, booster vaccines may not be inhibited by such exposure, but will have to improve upon protection induced by BCG and NTM. Modeling NTM exposure in preclinical animal models is extremely difficult, as it is likely that route, dose, NTM strain, and NTM virulence varies by age and geographical location. We need to measure NTM-induced immunity in the ongoing clinical vaccine trials, but such studies are limited by the lack of immunological tools with which to measure specific NTM-induced immunity. Attempts to identify NTM-specific antigens and NTM-specific epitopes within the immunodominant antigens currently included in subunit booster vaccines have been made, but are not easy due to very significant genetic sequence overlap between NTM and *M. tuberculosis* complex organisms [8].

This work takes us one step forward in the aim of developing an effective tuberculosis vaccine regimen for global use. Understanding why BCG works in some settings and not others allows us to optimize BCG administration. The development of appropriate immunological tools with which to quantify NTM exposure would allow us confirm or refute the role of NTM in explaining the variability in efficacy of BCG, and furthermore to use such tools to optimize the development of more effective and more consistent vaccination regimes.
